# Connected: Recommendations and Techniques in Order to Employ Internet Tools for the Enhancement of Online Therapeutic Relationships. Experiences from Italy

**DOI:** 10.1007/s10591-017-9439-5

**Published:** 2017-11-02

**Authors:** Gianmarco Manfrida, Valentina Albertini, Erica Eisenberg

**Affiliations:** Centro Studi e Applicazione della Psicologia Relazionale, Viale Vittorio Veneto 78, 59100 Prato, Italy

**Keywords:** Online psychotherapy, Group chats interventions, Online sessions, Psychotherapeutic emails, Advantages and risks of communication technologies, Therapeutic relationship online

## Abstract

The article explores the different types of therapeutic relationship that can evolve both on- and offline, thanks to the use of tools, such as software and applications, which enable therapists and patients contact outside of the traditional setting. Given the premise that it is practically impossible today to maintain a relationship without the use of internet and telephones, it becomes necessary to question the ways in which the online space can become a useful extension of the therapeutic setting. The authors, starting from a consideration regarding the specificity of the online therapeutic relationship, analyze the best ways to use text and email messaging with patients. Furthermore, specific interactions via group chats are presented, for example, to coordinate a therapeutic team involving several professionals. Further, video chat settings are discussed through a clinical case presentation. Lastly, the therapist’s management of social networks is debated, underscoring the importance for the therapists that his or her online identity be consistent with the offline image which patients are introduced to in the traditional setting of the therapy room.

## Limits and Utility of Online Therapeutic Relationships

According to Marian Sandmeier (2016), a recent research on Apple’s App Store yielded the existence of 1490 programs designed for anxiety reduction; 2193 to help with interpersonal relationships, and 948 apps to provide support for depression. Most of these apps draw upon cognitive-behavioral principles, others provide support either through self-help groups or through psychotherapist-managed groups. “We are sure that these apps cannot replace face-to-face therapy, or resolve personality problems which originated in childhood, nor can they help during severe crises such as divorce, illness, loss of a loved one, and even in non-symptomatic occasional situations such as repeated and frequent panic attacks, or obsessive compulsive disorders” (Sandmeier 2016). Rather than worry about the competition generated by these apps, psychotherapists who are not very interested in social networks and technology should ask themselves whether certain aspects of their professional activity could be improved by using—with psychotherapeutic skill—the available information technology for distance communication. Relational therapists especially, coming from a communication based approach (Watzlawick et al. 1967), should possess the skills to analyze the communicative characteristics of new technologies and use them to increase treatment effectiveness.

The possibility of keeping an open communication with patients through online technologies may have diverse effects, as:

- To provide support for severe, urgent or critical situations, outside of the time limits imposed by live sessions or telephone conversations;
- To extend interventions beyond the duration of the session itself, by decoding the meaning of the message received within the context of the therapeutic process and responding in the appropriate way;
- To reassure the patient regarding the therapist’s availability; messages arrive immediately or once the phone is turned on, without forcing the therapist to respond immediately or to interrupt sessions or other activities;
- To extend the power of the therapeutic relationship beyond the time limits of the session; during difficult moments, patients can feel encouraged, supported, contained, and assisted by their therapist wherever the latter may be;
- To reduce the frequency of sessions, reducing the cost for patients;
- To protect the therapists’ autonomy as he or she can decide whether, when and how to reply to a message. Therapists can take the time needed to reflect on a message, possibly even metacommunicating, and keeping in check a patient’s potentially excessive intrusiveness, or tendencies to act impulsively.

The aim of this paper is to illustrate aspects of online communication, as an instrument, which responds to the described purposes, within the context of the therapeutic relationship in systemic relational therapy.

## Online Relationships and Online Therapeutic Relationships

In this age of person-to-person networks, virtual relationships have been the object of several criticisms, yet the majority of scientific research seems to discredit this widespread attitude, underscoring that participation in online relationships coincides, in many cases, with a greater ability to relate in presence (Rainie and Wellman 2012). New technologies are a tool, which, if well managed, can facilitate social (and thus therapeutic) interaction: it is therefore worthwhile to create a space for reflection and consideration on how new technologies can be wisely integrated into our personal and professional relationships.

A study on the use of online messaging by teenagers in the United States (Lenhart et al. 2010) pointed out that, for the younger generation, text messages are not considered “writing”, rather they are equal to a “conversation”, indicating that, to date, the difference between written and oral communication is slowly disappearing, at least on the mobile phone. Research on what compels young people to communicate online (Papacharissi and Rubin 2000) highlighted five main motives: maintaining relationships and keeping constantly in touch with friends, even with those that are physically distant (Valkenburg and Peter 2007); meeting new people (Leung 2001); social compensation, compensating problems in real life communication by online socialization (McKenna et al. 2002); social inclusion and the need to belong to a group (Mitchell et al. 2003); and entertainment (Ferguson and Perse 2000).

Meaningful work (Baiocco et al. 2011) compares two opposing explanatory hypotheses to interpret the relationship between online communication/socialization and psychological well-being of preadolescents and adolescents: disengagement theory and stimulation theory. Disengagement theory states that online communication has a negative impact on psychological well-being because it consumes time, which could otherwise be devoted to improving the quality of existing friendships, and stimulates the tendency of young people to establish short-lived and emotionally insignificant relationships with strangers. The underlying assumption is that the internet is a poor “virtual substitute” for face-to-face communication and provides some compensation for the difficulties experienced in off-line relationships (Lee Saunders and Chester 2008).

Stimulation theory holds, on the contrary, that online communication fosters the enrichment of the individual’s relational context and promotes opportunities for growth and adaptation. Research has shown (Valkenburg and Peter 2007), for example, that the time spent with instant messaging predicts children’s quality and quantity of interpersonal relationships and their psychological well-being. Investigation is still being brought on, but stimulation theory is getting more confirmation (Laghi et al. 2013).

In psychotherapy is online communication a form of disengagement, keeping a distance between patients and therapists, or is it stimulation, connecting them more strictly? Although there is still need for further research and for a broader discussion, there is no doubt that—as therapists—we observe that the stimulation theory is by far more appropriate to describe the relational setting than is the disengagement theory. The possibility of communicating with technological means has already resulted in the introduction of new and sometimes vital elements into the therapeutic relationship. Selvini Palazzoli et al. (1978) created a ‘caller checklist’, underscoring the importance of the initial contact with the patient and the need to collect data and structure the interview from the very first telephone contact with the family. It is impossible to avoid the impact of technology, and it is therefore fundamental to explore in depth how relational aspects of the settings are affected via online communication.

## Therapeutically Reading and Writing WhatsApp, Text, and E-Mail Messages

What is the difference between a text message (an SMS), an MMS, an iMessage, or a WhatsApp message?

SMS/MMS messaging enable one to send text messages and photos to other devices using phone credit (Manfrida 2009); iMessages allow one to send text messages and photos to other iOS devices via Wi-Fi without charge; the same goes for WhatsApp and similar messaging apps, with the added difference that these apps are available for iOS and other operating systems such as Android, Windows Phone, and BlackBerry.

According to data published by AGCOM, the Italian Authority for Telecommunications (2016), WhatsApp has overthrown old text messages, leading to an incredible drop in the use of the latter. In 2016, in Italy traditional text message usage dropped by 27.7% when compared to the previous year, reaching the quota of 17.8 billion messages resulting in a drop of −75% when compared to the maximum level reached in 2012 (72.2 billion text messages sent). This downward trend has been constant for 4 years: sending standard text messages via phone has now been supplanted by online messaging. Today the use of SMS is mostly limited to businesses or for exchanges, which require some level of formality whereas iMessages, WhatsApp messages and the likes of are used for personal and informal communication with acquaintances. Given that they do not rely on the availability of a wifi network, traditional text messages actually have a greater guarantee of delivery; usually contact with a professional is initiated via text message, even when help is sought for a child—who would spontaneously use WhatsApp—parents looking for a specialist will rely on traditional text messaging: once therapy has begun, in our experience the use of text messages is often replaced by WhatsApp messaging. On the other hand, traditional text messages protect the therapist from the embarrassing problem created by WhatsApp sharing information about the user such as “last seen at ...” or “message read at ...”. In fact, once the WhatsApp message has been opened this is signaled to the sender (unless the user disables this function), therefore the time passing between the therapist reading the message and a reply being sent could be interpreted in relational sense as an immediate guaranteed availability or substantial lack of interest that may affect the therapeutic relationship. Choices such as disabling the message receipt notification, when to open a message, when to read it, and when to reply to it, acquire therapeutic value and imply an acceptance of responsibility on behalf of the therapist, which should be managed within the relational context, consistently with the therapy goals. Should we exclude our clients from the reassuring possibility of seeing that we have indeed received their message or will this entail a constant commitment throughout our day? Sooner or later, however, we will have to reply to these messages and give them our full therapeutic attention.

On WhatsApp it is possible to add a profile picture; while these images and pictures can give us some information regarding our patients, they can also—inversely—provide our contacts with information about us, therefore therapists must be extremely careful when choosing a profile image, lest it might interfere with a good therapeutic relationship. For example, using a picture of one’s happy family on holiday can have an impact on the relationship with less fortunate patients; pictures of ones’ children can create problems for patients who might be trying to have some; using funny images can expose one to the risk of ridicule during moments of conflict that may occur during therapy.

The way in which WhatsApp, text, and email messages are written should never be considered casually, this concerns messages that are being received or sent by the therapist. A text written in all caps or one that is interrupted by an excess of ellipsis seems to suggest a diagnosis of histrionic personality, if only for the choice of characters: thus a message may already contain a diagnostic suggestion (Manfrida and Eisenberg 2007; Manfrida 2009; Manfrida and Albertini 2014). An unsigned message sent to the therapists suggests that the patient expects to be immediately recognized or, even better, that he or she is unique for the therapist; if so, responding with a message that points to the lack of a signature, immediately restores the reality principle to the therapeutic relationship, in addition to sparing the therapist the effort of identifying the sender by cross checking his or her phonebook. The fact that the sender’s name can be often identified by the phone should not detract from the significance of this observation, because patients should not assume that they are recorded in the therapist phone directory and, in any case, it is good practice in a professional context, to sign one’s messages: indications to sign one’s messages are always given to patients at the first visit.

Sometimes messages may contain inconsistencies and contradictions, which can be reported immediately and examined later in therapy by the therapist, in order to activate the patient’s curiosity and to train him or her in the skill of psychological investigation.

The “expressive” use, often rhetorically structured in order to gain impact, of messages is also indicated by the use of punctuation, as in the following example, in which it is clear that the patient really wants to communicate her news in a thrilling fashion:

Patient: *Hello, It’s Cristina ... I have some news... I have been pregnant for approximately 8 weeks. Clearly I made a mistake in my calculations!*

Like patients, therapists also have to make some stylistic choices when replying to a message, in order to confirm and reinforce it with persuasive elements. For example, in replying to a message cancelling an appointment the therapist may choose to adopt a neutral tone, or a concerned one, or even an indifferent one, all depending on the significance, which he or she ascribes to the cancellation, the moment of the therapeutic process, and the intention with regards to the intervention.

Using a patient’s name may convey a more “personalized” message, giving more warmth to the written word. The placement of the name in the sentence can also express different shades of meaning: e.g., “*John, you did something stupid*” is more friendly and less accusatory than “*You did something stupid, John!*”, where the proximity to the end of the sentence, especially if there is an exclamation point, seems to imply a scolding rather than consoling solidarity. Solidarity can also be enhanced by the use of punctuation: “*John, you did something stupid ...*”.

It can be stated that sentence structure, word distribution, punctuation, the way in which sound resonates within the reader, and other resources also give written messages those connotative aspects of meaning which, in the pragmatics of communication, are ascribed to the analogic component of language (Watzlawick et al. 1967).

Therapists should always sign their messages, in full or with their initials; this is not a simple matter of form or good manners. Closing a message with “Dr. E. (initial) B. (full surname)” or “GA (abbreviation)” represent context markers, indicating that it is a message within the context of a professional relationship, reducing the risk of excessive familiarity.

If therapists feel that they are receiving too many messages, or should they wish to emphasize the unequal balance of the relationship for therapeutic purposes, they can use these messages in several ways: it is possible to respond with a message that underscores the excess of incoming messages; choose not to reply; choose to reply only after length of time that would suggest to the patient that he or she overdid it; or save the messages and use them during the next session to work on them within the therapeutic relationship. The therapist’s choice of the appropriate words to generate the desired psychological effect in the patient also requires a significant degree of commitment: a simple encouragement can be very different if expressed with a “*Bravo!*” or a “*See you soon*.” A joke or a witty comment can confirm mutual understanding in a specific situations, for example: “*Congratulations, a prestigious result that will give you credit and ... customers!*”. In other circumstances, it is appropriate to use more generalized expressions, e.g., “*I’m glad you have had such a good result*.”

Text messages can be enriched by the use of emoticons or emojis (Table 1); these more or less cheerful icons are part of a shared code of communication of emotions and moods. Emojis are usually added to messages, to specify the emotional status of the sender in order to avoid the risk of a misunderstanding. The words “*I hate you!*” take on a different meaning when they are followed by a smiley face , thanks to the analogic level prevailing over the digital one not only in oral but also in written communication (Watzlawick et al. 1967).

**Table 1:**
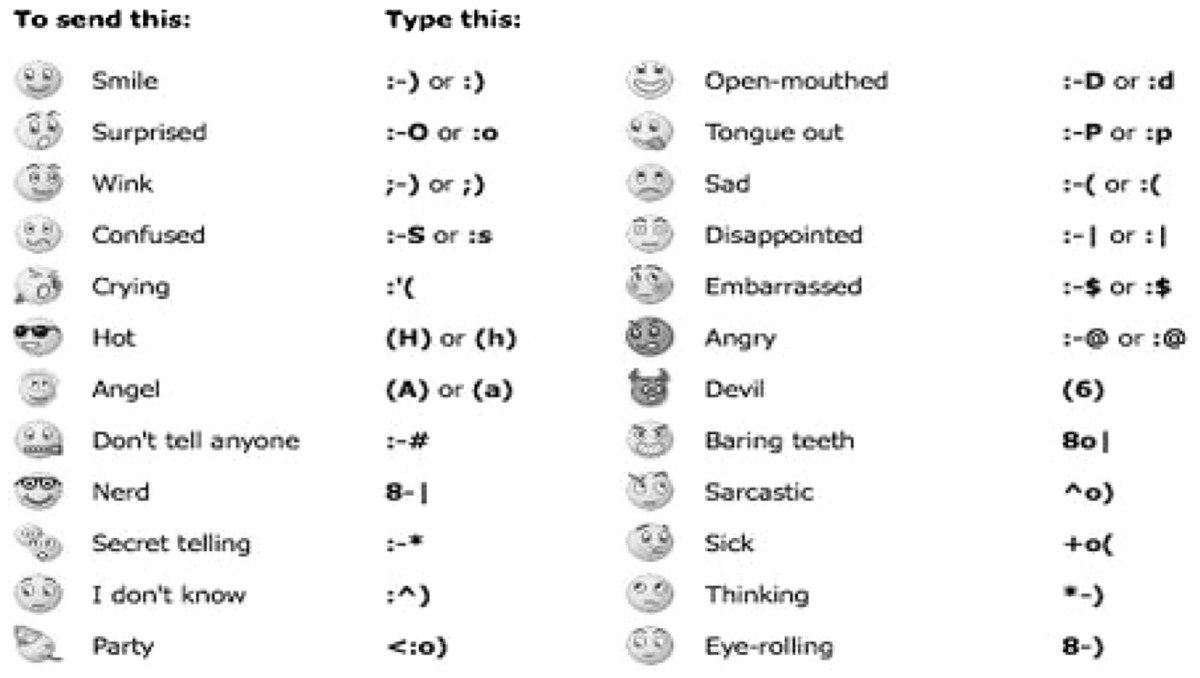
Using smilies (emoji). Source: http://graphicsheat.com/emoticons/#sthash.SbzQvk3i.dpbs

When texting it can sometimes be difficult to convey feelings and emojis can help solve the problem. Some people prefer to use characters (shown on the right hand column in Table 1), to create expressive images, like :-) or :-(. “*You’re a wretch!* :-)” is more reassuring than “*You are a wretch!* :-(:-(:-(8-[“. The creative ways in which emoji and characters are used underscore the importance that humans attach to the relational aspect of communication, which is assimilated from early childhood in the social process of building a shared reality.

## Some Examples of Exchanges Via WhatsApp

All names have been modified and circumstances have been altered in order to preserve privacy.

### First Example: Francesca

Francesca is a 31 year old nurse, at her fifth therapy session. She has a diagnosis of depressive disorders and borderline personality disorder, and has made one suicide attempt by overdosing on medication. She lives on her own; the suicide attempt followed the end of a romantic relationship with a highly problematic man who was unfaithful, violent, demanding, and despotic.

During an earlier session, which took place 2 days prior this exchange, Francesca reported that Richard had approached her again, and she felt severe emotional difficulties. During this initial phase of therapy, the therapist’s goal is to become a solid reference point for the patient given that this early in the process it has not yet been possible to establish an alliance that would prevent the recurrence of attempts at self-harm. An alarming factor is represented by the time of the first message and from the mention of the day spent in bed (meaning, she has skipped work and has been home alone all day). The therapist’s messages respond to Francesca’s request of not being left alone; at the same time continuing the work initiated during the last session, in which they agreed to change the image of Richard as a unique prince charming, and to look in the patient’s history and personality for the causes of her suffering, in order to reduce her responsiveness to uncontrollable external stimuli such as Richards’ coming and goings.

Francesca, Wednesday, Feb. 1st, 11:40 p.m.:

- *I always felt I was outside of a social life. With him I felt normal. Even being cheated on is normal ... Pride has little to do with it...*

Therapist, Thursday, Feb. 2nd, 10:00 a.m.:

- *So competition is not stimulating for you right? Come on, Francesca ... do not play the ‘crazy in love’ part.*

Francesca, Thursday, Feb. 2nd, 10.15 a.m.:

- *Pride matters ok, but it’s marginal. It’s loneliness that generates these anxiety crises so I do what I have to do to not be alone. If I lose him, I go back to being alone.*

Therapist, Thursday, Feb. 2nd, 11.05 a.m.:

- *It seems to me that Richard’s companionship is too demanding and uncertain for you ... But do as you wish.*

Francesca, Thursday, Feb. 2nd, 6:56 p.m.:

- *I feel as lonely as I did a year ago before I met him. I’ve been in bed all day. What can I cling to?*

Therapist, Thursday, Feb. 2nd, at 7.10 p.m.:

- *You know very well that in the meantime you can cling to me, which is why you call me and text me… and that’s good, you can rely on me, Francesca. Then we’ll see.*

### Second Example: Claudia

Claudia is 22 years old, she began therapy 2 years before this exchange because she suffered from bulimia; once this problem was solved, therapy was addressed to help with growth and development problems that had blocked her academic and affective life. The message follows a period of supportive activities aimed at her returning to attend university. The therapist’s message aims not only to celebrate the results obtained by the patient, but also to make her gain confidence in the qualities of character in which she feels lacking. At the same time, the therapist discounts his part in achieving the result, accepts the affective request through the “hug” but replies with a moderating “regards”. In addition, the message implicitly confirms the next appointment, warranting that there is no risk that the therapist will withdraw if the patient makes progress.

The message is therefore aimed at providing therapeutic support, but goes beyond praising the single positive result and extends its impact towards personality reorganization.

Claudia, Saturday, Apr. 1st, 6:46 p.m.:

- *I also passed French and the teacher who had failed me the other time actually complimented me this time! I’m really happy. See you on Wednesday. Hugs.*

Therapist, Saturday, Apr. 1st, 7:05 p.m.:

- *Excellent Claudia! I am really happy for you. You’ve shown ability, determination and willpower, and been deservedly rewarded. See you on Wednesday. Kind regards.*

### Third Example: Luigi

The connection via message was agreed upon in a session before the patient’s departure; this enabled Luigi, who suffers from panic attacks, to face a plane trip with his girlfriend and some other friends. The success of this initiative can contribute to an increase in self-confidence and reduce the risk of recurrence of panic attacks, and also avoid the onset of problems within the affective relationship and the social reference group. The response confirms the therapist’s protective interest, without putting too much responsibility on the patient, thus restoring the experience within a normal framework.

Luigi, Sunday, Mar. 13th, 2:05 p.m.:

- *Dr. good morning, this is Luigi, I have just arrived at Budapest Airport ... everything is fine here ... the trip went well. Thank you so much! Greetings!*

Therapist, Monday Mar. 14th, 9:20 a.m.:

- *Luigi, I’m glad that the trip went well. Relax and enjoy yourself. See you soon.*

Some examples of common WhatsApp messages by topic:

### Medication

Patient: *If I were to increase the sertraline, when should I take the second tablet?*

### Cancellation

The reply is friendly, while at the same time underscoring that it is not up to the patient alone to decide the date of the next appointment.

Patient: *This is Tom, I am sick. I’m fine for the 15 if there are no problems. Thank you.*

Therapist to patient: *Tom, I’m sorry you’re ill. Call me before 11.30 am or from 1:00 pm to 1:45 pm and we can schedule our next appointment.*

### Information/Control

Receiving messages which concern difficulties and progress, enables the therapist to monitor what happens in the interval between sessions. Therapists respond with participation, providing support and assisting during the hardest moments, while reinforcing positively the well-deserved successes.

A. Patient (1 week after the first meeting): - *Good morning, there are no improvements, I feel down and I’m afraid that I will not make it.*

Therapist: *I understand your uncertainty and discouragement, but you know that you are loved by those around you and there’s no reason to feel like a lost puppy in a forest of wolves. Also, soon the meds will start working. Hang in there!*

B. Patient: - *Good morning, I wanted to say that everything went well in Caserta. I apologize for sending this message with a few days delay*.

Therapist: *Marcella, congratulations. I am very pleased.*

### Psychotherapy

A. The therapist responds to the request for comfort and support, and also lays the grounds for the next session, inviting the patient to a greater commitment and initiating a discussion concerning the therapeutic relationship itself which should be understood as a support or as an alliance for change.

Patient: *Good evening Dr.! I am a bit down and I would like to receive, if you can, a comforting message. My situation with Mauro has not changed. Thank you and I apologize.*

Therapist: *Luciana, you have all the comforting and support I can provide, but first I would like to know why you did not come to the last session on Feb. 10th, what happened to you?*

B. The therapist in the next message detects the dramatic tone accentuated by writing in all caps, which contrasts with the option the patient gives herself of deciding in the morning whether to come at the appointed time or later. The therapist rejects the patient’s attempt to drop all responsibility while at the same time guaranteeing his availability; the therapist also creates the premises to discuss—in the next session—the patient’s need to actively start treatment for her alcoholism instead of pitying herself and demanding pity for her incapacity. The therapist deliberately chooses to respond briefly, using only lowercase letters, to emphasize the exhibitionistic component of the patient’s message.

Patient (the different letter style reproduces the original): - *I* *HAVE BEEN IN THE GRIPS OF ANXIETY ALL DAY. IN THE END I COULDN’T TAKE IT ANY MORE AND I GAVE IN TO MY ‘SELF-TREATMENT’ BY DRINKING LIQUEUR. I CRIED SO MUCH. I AM ASHAMED. I WANTED TO TELL YOU.*

*PS I HOPE TO GET SOME SLEEP TONI* *GHT AND BE THERE TOMORROW BY 10.30, IF NOT I WILL CALL YOU FOR THE 1:00 PM GAP.*

Therapist: *Set your alarm clock, Anna, the 1:00 pm gap is no longer available.*

C. The following is a very brief exchange that implies an ironic reflection on the recurrence of a problem within the therapeutic relationship and which could take place in any therapy session.

Patient: *There must be a reason if I always get sick 2 days before an exam ...*

Therapist: *I am beginning to think that myself ...*

Patient: *That’s encouraging...*!

## E-Mail: Specific Aspects of Therapeutic Communication by E-Mail

Unlike WhatsApp and text messages—the exchange of which is perceived more as a conversation,—e-mails given their usually greater length are experienced more as letters and are therefore suited for situations in which therapist and patient, given a physical or temporal distance, opt for an exchange of letters rather than face-to-face meetings. While all the general comments made about texting can also be applied to e-mails, there is more room in the latter for the patient’s narrative and for the therapists’ reply, while there is less space for brief comments, which—in text messages—refer directly to the sessions attended. In the course of therapy, it is possible that face-to-face sessions are supplemented by text messages, WhatsApp messages, or e-mail exchanges, according to the different expressive needs, which call for different modes of communication.

If face-to-face sessions and text messages can be challenging for the therapist, an excessive number of e-mails can become really difficult to manage: longer messages must necessarily be limited to infrequent occasions, and any excess should be discussed during sessions to restore them with their therapeutic significance.

## An Example of Text Message and E-mail Therapy

We describe a clinical case which was carried out with the support of new technologies, specifically text and e-mail messages (Written with the consent of the client after editing of all details making her recognizable).

The patient is a 43-year-old woman; she works as an accountant, divorced, with a 15 year old son and two dogs. She has been in therapy for over 2 years, with session frequency every 2 weeks. Initially she sought help for depressive and anxiety disorders with self-destructive ideas, and then she began to work on her personality.

The patient presents a split situation: on one side, she is responsible, serious, maternal, reliable and precise; on the other she acts out risky behaviors in terms of mismanaged sexual promiscuity, sporadic but intense use of stimulants, and an elevated exposure to unnecessary dangers. She introduces herself as Mary; in the course of treatment, it emerges that her full name is “Mary Magdalene.” “Mary” is the one constantly looking for a unique romantic love to be lived with adolescent transport; in addition to assisting her elderly parents and providing for her son, Mary has also adopted two stray dogs. “Magdalene” is her sinful, uninhibited, part; the brilliant seductress, proud of her freedom and experience. This personality split reminds anybody in catholic Italy of Holy Mary and the repentant Magdalene from the gospels.

In addition to the fortnightly meetings, there are periods in which, due to a holiday break or to the therapist’s absence, therapy also involves contact through text messages and email. The goal is to maintain a level of involvement in the relationship with the therapist, which will facilitate working on personality.

July 10th, 9:29 a.m. Text Message from Maria

*Forgive me if I bother you again but I absolutely need your immediate advice: the person of whom I spoke yesterday is a colleague. He is 49 years old and unable to create a lasting relationship because (his words), he is constantly divided between the desire for sex, love, and freedom. … The fact is that there is between us, at least for now, a very intense understanding, and last night, Doctor, in bed, I felt a complete oneness with this person. We made love in a completely enveloping way, talking, laughing ... And he too felt these same feeling, even though he continued to say that he knows himself. If yesterday I was in a crisis it is even more so now. I sent him a message and he replied ‘big kiss’. What should I do? Help me please. Maria.*

July 10th, 9:35 a.m. Text from Therapist

*Can you take it VERY lightly, and settle for a few encounters and little emotional investment? The only way to get the attention of this kind of person is by running away, showing that you don’t want to invest in this relationship either. But can you do this or is this the usual trap for Maria? Please consider if the game is worth the prize, anyhow we will talk about it. gmm.*

July 18th, 7:46 a.m. Text from Maria

*The millionth disappointment. There was no point in continuing to see each other but the problem is, he really got into my head in an incredible way. with this person I am in a full blown crisis. Anyway, the biggest problem is that I do not feel understood by anyone, not even by you, you think that I jump into every relationship because I feel small and ugly… That’s not true, I just have so much love to give and i really want someone by my side. I really feel misunderstood.*

July 18th, 9:56 a.m. Text from Therapist

*Don’t act like a rebellious or misunderstood adolescent with me too, I do not underestimate your suffering or your anger, I am trying to treat you like an adult, will you allow me to? gmm.*

July 18th 10:00 a.m. Text from Maria

*Honestly, I have the impression that you are not treating me like an adult but like a silly child, to say the least…*

July 18th, 10:05 a.m. Text from Therapist

*You see me as too harsh… gmm*

Continued therapy will necessarily encounter holiday breaks; for patients this can represent a brief yet painful and difficult separation. Given the unstable situation, and the recently initiated work on personality, the therapist gave Maria his email address to contain any potential emergencies. During this break, one of Maria’s dogs is killed in an accident, renewing feelings of loneliness and loss, to which she reacts by adopting another stray just a few hours later.

In the following email, sent on August 18th (the last session had been scheduled on July 21st) both the content and the style of writing reveal aspects of anxiety, confused and accelerated thoughts, histrionic attitudes—for example in the excessive use of ellipsis.

August 18th. E-mail from Maria

*I have collapsed both physically and psychologically… another dog … I wonder what I thought it could give me … And then … She too is gone … Under a car … atrocious pain … Doubled over in the middle of a road crying for my little doggie who wasn’t there anymore …. I immediately got a new one at the shelter, a dog bit me …*

*Ernesto was getting worse … His wife was getting closer to him … He would have preferred to have me there but if the wife was there they wouldn’t let me in … He did not wait for me … I saw him once he was dead … I held him … I kissed him … I bought red roses for him …. They threw them away in front of my eyes, they called me a whore, they kicked me out of Ernesto’s house… I wasn’t there when they closed the casket, I wasn’t there when they buried him…*

*I have such immense pain inside me … I don’t think anyone can understand...*

*I put all my energy in my job … lots of work, lots of sacrifices, lots of words because I can’t shut up, so much anger inside me …*

*I went out with many men...*

*I went to Trieste for a course with a coworker…I also went away for a weekend with one guy to Turin…*

*All this until 15 days ago … I meet this last coworker … Nice guy … We really hit it off and we spend hours talking … we make love with unique transport … not sex … Love …*

*I drop everything else…. But he doesn’t want a relationship, he says he’s a womanizer and that he doesn’t want ties… I breakdown, I am in pain, I tell him … I don’t want to see him anymore …. But after 2 days we meet again, we have a drink, then he comes to my house but it’s not the same anymore … He realizes that I’m sad … it’s true. I am more detached … He too is sullen … He says he’s got the blues … and that brings us to today…*

*What the heck am I doing…*

*My son is growing … what kind of mother am I … can I give him any serenity?*

*I realize that I am unsatisfied … these races, constantly looking for who knows what…*

*I got another dog… Happiness in the house but even more concerns, fatigue, the desire to run away…*

*Chaos … Just chaos … Exhaustion … So much exhaustion … I need help … A lot…*

*Maria.*

August 18th. E-mail from the Therapist

*Dear Mary Magdalene,*

*You made a hectic summary of your story, and it’s easy to see the feelings behind that: hope, anger, fear, your provocative brazenness, tenderness, strength… These are all aspects that belong to you, none of them are worthless, but with all the contrasts you carry inside it is no surprise that your wishes remain unsatisfied. One look at your email is enough: it is a vortex that leaves one breathless and stunned just by reading it, I can’t imagine what living it might feel like. I am not saying that you always find the wrong guys; I do say that if they are right for a part of you they are not for the other, and that you would be better off working on that division you feel inside, between the child longing to be loved freely, as if by a parent, without having to commit oneself, and the daring, rebellious, offbeat teenager screaming “I am mine and I do what I want”. I like both of those faces, both Mary and Magdalene, but it would be nice to have them together for once! Instead it seems that when you feel anxious you become extremely active, in order to gain validation of your skills, be they professional, seductive, sexual, parental, relational, adoptive of dogs, and so on and so forth. You can gain satisfaction, but it’s only temporary if a part of you is nourished while the other starves.*

*Now you are angry with me… because I tell you not to run after this man who clearly drew the line from the very first moment you met? Does this mean I am so cruel that I wish to deprive you of a legitimate dream, or perhaps that I care for you and I would like to spare you a pointless nightmare? And in any case, if a man tells you that he is unfaithful, wouldn’t it be better to reply “really? How fortunate! I am like that too, so we won’t be getting in each other’s way”—so, if he will wish for more he will have to step up and chase after you, and if he does not do that, OK, he’ll be a fun playmate until you find what you need somewhere else?*

*There is something in your anger towards me that I am not sure about, Mary Magdalene; is it because last time we spoke we shifted the attention to your childhood? Because I spoke of your parents? Or is it that, with me too, you alternate between wanting protection and defiance? Your two faces are also present in the relationship with me, and this exposes you to the risk of running to stand still, of never being present in the relationship, because you are constantly switching between Mary and Magdalene?*

*I like you, and I really wish to help you; will you let me do it, even at the cost of one of your extremes being disappointed? On the other hand, the first necessary exercise for you to find a partner that will satisfy both extremes is to be able to be fully present in therapy, with yourself and with me. Can you do that?*

*Especially now, during this holiday break, I assume you must feel abandoned by me too, so you must be angry for this umpteenth disappointment at the hands of the umpteenth man, the undersigned, who is apparently there and then suddenly disappears? With some confusion, the shifting of roles and of emotions, which is rather common and useful if they can be identified and worked on in treatment. Even in an email, like this one! Read your email again, be a detective, or a psychologist… look at all the elements that allow me to talk like this … and when we meet next time we can clarify all! See you soon, Gmm.*

## Conclusive Session Feedback via E-Mail within the Family Therapy Setting

It has long been a common practice in Italy to end family and couple sessions giving a conclusive feedback, undiscussed with clients on the spot: Selvini Palazzoli and her Milan group (1978) described very well this practice, which has been brought on by many Italian and foreign therapists.

At the Center for Family and Couples Therapy, in Prato (*Centro Studi e Applicazione della Psicologia Relazionale*), where we work with the use of the one-way mirror and videorecording sessions, it has become our policy to send each participant over the age of 12 the conclusive feedback we give at the end of each session. Because sessions usually occur on a monthly basis, sending the patients an email with some kind of summary of what took place during the session is also a way of keeping the patients involved in the process during intervals. It is also possible, with the clients’ consent, to share the session feedback with the therapists who are seeing family or couples members individually, in order to coordinate contents and timing of the therapeutic interventions, both on the individual members and on the couples or family systems.

### E-mail Feedback After a First Couples’ Therapy Session

*In your narrative there were moments of heartfelt and deep suffering, and other moments in which the setting resembled that of a soap opera, starring a hypermacho male and a sweet and whiny female lead. Title of the soap opera: executioner and victim. You are wonderful actors, but your drama did not fully convince us, even if we can perceive the suffering in both of you. To understand your present situation, we believe it is necessary to look at your history and ask ourselves what was so special of Rossana at 21, which enabled her to rope in a 27-year-old confirmed bachelor, who had plenty of experience with women, drugs, and the world in general. Why choose her to bring about such a dramatic change in his life, which would also lead him to move to another region?*

*And Rossana, at 21 she was looking for prince charming who would take her away from her family and from Naples, but why identify this prince with a man who is better suited for the special ops? She also wanted affection, not just muscles… 15 years have passed and a lot of things have surely changed in the interval. Now, counting your children, there are four of you, and things are very different from when there was just the two of you. Rossana has also made a lot of progress in her work and in individual therapy, she is less needy of special protection now, and this creates a distance with Roberto. Regardless, the fact that your life situation and your beliefs have in part changed does not mean that there wasn’t a special attraction at the beginning of your relationship, and that attraction may surface again if you can change some of the rules of the way you are together. One of these rules could be: less Rambo and less Little Matchstick Girl. Let’s change the screenplay and perhaps this will enable you to find each other again, a little different but similar to the way you were.*

*We will see you again on Feb. 16th at 5:30 p.m.*

### E-mail Feedback After the Sixth Session of Family Therapy

*We have made a great discovery today, which we hope you will all treasure. You are a family, specifically, the Flintstones! Giovanni is Fred, the burly caveman dressed in leopard skin, who hollers out “Wilma, my club!!!”. Luisa is Wilma, well-mannered wife, who will not spare the club if she gets angry. Mariangela is Dino, the over friendly dinosaur pup that jumps on Fred every time he steps into the house. And then there’s Pebbles, their daughter, always on the sidelines. Despite the leopard skin and club, Fred often reveals himself to be an overgrown clumsy child with a kind heart, with a tendency to get himself in sticky situations which Wilma has to save him from. This is possibly why Fred is always afraid of Wilma and of her anger and he only feels comfortable with his buddy Barney. I don’t know who your Barney is, perhaps your father? In your home there is a situation now for which nobody feels appreciated or loved and everyone does anything possible to be even less appreciated and lovable. In truth, Fred—pardon—Giovanni, would expect to be loved and cared for in the same way he was by his family of origin. Because Luisa, Mariangela and the rest of the world are not his parents, but they are people who need him, they are reluctant to treat him like a spoiled child; Giovanni becomes angry, he whines and complains a bit, and when he does, the others respond along the same lines.*

*Even though we understand this better now, and we hope you do too, we don’t know if you will be able to set aside your demands to meet each other’s needs, but we genuinely hope so, because the Flintstones have a lovely family, whereas your life often resembles a nightmare.*

*We will see you all again on February 16th at 2:30 p.m.*

## Therapists’ Groups on WhatsApp and Email

WhatsApp allows one to create chat groups; this can be a useful tool to quickly communicate with a group of people, thus coordinating therapy interventions among the different therapists working with members of the same family (or somehow related to each other). If this—on the one hand—exposes one to the risk of excessive uniformity of interventions, it also allows therapists to stay in touch and help each other during critical moments. Furthermore, group chats enable participants to share information which can be priceless when patients are reticent: even if the data obtained from colleagues cannot be used within the sessions, it is helpful in leading the discussion towards these unwanted topics. Finally, groups can represent a resource by providing emotional support for those therapists who—at times—may feel uncertain or may be facing a special difficult moment, and they can be reassured by their colleagues’ participation in the process. The *sine qua non* condition that must be enforced is the utmost respect of patient confidentiality, getting their consent and limiting participation to the group discussion only to those therapists who are directly involved in the case work.

## An Example of a WhatsApp Group Chat Among Three Therapists

In the following case, a family of three is being followed in individual therapy by three different therapists. The three patients have been referred to individual treatment following the advice of a family friend who shares, with the therapists, a relational systemic training.

Dr. Jim is seeing Terry who is the wife of Victor (in therapy with Dr. Gail); Bernard, the son, is being seen by Dr. Zack. Terry and Victor’s marriage alternates moments of intense attachment to moments of indifferent and resentful detachment. Victor is the last one to have requested treatment, mostly because of the pressure from his wife; he is very withdrawn, he does not talk about his activities or of his family history with anyone, not even with his son. A reticent man, who was capable of checking into a hospital for surgery without anyone knowing, and showing up a few days later at the yacht club.

What follows is a transcript of the messages exchanged in a group chat by the three therapists:

Sept. 14th, 11:39 am, Dr. Gail: *I have bad news: Victor (husband) isn’t answering the messages I have sent him. At this point I feel that it will be impossible for me to involve him. I am terribly sorry about this because he was improving. I trust in the old saying “never say never…” but for now I have to sign off. If you allow me to, I will continue to follow the development of this case through this chat. See you soon.*

Sept. 14th, 3:35 pm, Dr. Jim: *Dear Dr. Gail, Victor is a very difficult patient to involve, and you were able to do that… let’s wait and see what happens… Monday the 23rd, after seeing Terry, I will write in this group again. Working together is a great opportunity for personal and professional growth. Hugs to all!!*

Sept. 15th, 3:52 pm, Dr. Gail: *Wow Dr. Jim, you must have superpowers… Guess who sent me a message?*

Sept. 15th, 3:53 pm, Dr. Zack: *Victor, I imagine?*

Sept. 15th, 3:54 pm, Dr. Gail: *Exactly!*

Sept. 15th, 4:45 pm, Dr. Zack: *I knew it, you have managed to involve him and he is the type who will not commit himself completely to anyone but he will keep everyone… hugs.*

Sept. 15th, 4:50 pm, Dr. Gail: *That’s exactly how he is: he keeps everyone, even the sister that creates so many problems. I’ll let you know if I manage to finally see him. Hugs.*

Sept. 15th, 4:58 pm, Dr. Jim: *Dear Gail and dear all, I am so pleased by this news! I knew you had managed to involve him. You did build a therapeutic alliance with him. Waiting for the next updates, hugs to all!*

Sept. 24th, 2:35 pm, Dr. Jim: *Dear all, yesterday I saw Terry; Victor told her that because of his health problems (hospitalization, surgery, etc) he missed some sessions with Dr. Gail. Terry (wife) has urged him to go back to the sessions because when he goes he feels a lot better!! I will see her again next Monday, if anything comes up that might be useful I will let you know. Hugs to all.*

Sept. 24th, 2:49 pm, Dr. Gail: *If he doesn’t bail out, fingers crossed, I should see him tomorrow… talk tomorrow.*

Sept. 25th, 4:18 pm, Dr. Gail: *Hello group! I just saw Victor and we had a beautiful session. He told me that he has become a vegetarian, (meaning he really did get scared of dying) and this triggered a discussion on all his fears including the ones concerning his crazy sister. He said that he would like that in the future everything remain exactly as it is now and that nothing unpleasant EVER happen. I answered that bad things happen and added that he will be able to manage them as well as in the past and even better! Now he can fight alongside Terry and he acknowledged that she is a great support for him. I wrapped things up by saying that collaborating with his wife is the best way to make her feel loved.*

*I take nothing for granted with this man, so I am not sure that he will not bail out in the future… but I do have some degree of certainty that what I said to him today hit the mark, given that he left the session feeling definitely happy.*

## Sessions via Video Calls

Is it possible to conduct an entire therapy online, without there ever being a direct, face-to-face meeting between patient and therapist? Unless there are severe impediments, such as the patient being housebound due to illness, we discourage from a therapy plan, which does not include regular meetings in person. We are well aware of how difficult treatment can be, so it is important to note that a patient’s request for a long-distance treatment may conceal an attempt to remain distant, without ever entering the therapist’s study be it virtual or physical. The same concern applies to the therapist who can, subconsciously, search for a long-distance relationship as a protection from excessive involvement.

Even therapies with protocols that do not rely heavily on emotional and improvisational aspects still need a basic emotional involvement in order to be productive: simply seeing or listening to the therapist cannot replace that physical proximity which, itself, helps generate trust. In order to decide whether the therapist is trustworthy, whether the session is just a customised lesson in theory or whether it can be a life-changing opportunity, it is necessary to hear the tone of voice, see his or her expression up close, and perceive the full size of the therapist, not just that conveyed by a 4 or 7 inch display.

The physical presence is even more significant when treatment is aimed not only at reducing symptoms, but to the underlying personality disorder, in this case the therapeutic alliance is, *per se*, the most powerful therapeutic tool (Cancrini 2006; Smith Benjamin 2003).

Due to chance reasons (emergencies, temporary unavailability of the clinical setting, etc.) or to specific life events (patient or therapist relocating, illness of either party, long breaks due to holidays), it may be necessary at times to hold some long-distance sessions.

In such a circumstance, video conference tools might be a valid instrument to prevent the interruption of therapeutic work, and to warrant some kind of continuity in a way that is most similar to the usual one, avoiding the sequentiality of written messages and maintaining the feeling (illusion?) of a direct interview. Despite the undeniable convenience of these means, management of the online setting becomes an important issue. Online sessions are not a simple technical transposition of a face-to-face session (Mingione et al. 2016) since it acquires new communicative dimensions.

First and foremost, the scenario may be different from the setting that patient and therapist are accustomed to; within the practitioner’s cabinet, every element is framed within a professional context. In a video conference, one or both parties will surely be in a different context from the usual ones. There will therefore be many elements that will become part of the setting which may require special attention; pictures hanging on the wall (regarding the patient, these may provide a topic for the session, for the therapists, they may reveal aspects of their private lives which they would prefer not to share); lighting, images, photos of friends or relatives… not to mention household pets.

The therapist can enter within the patients’ world in a more direct way than what is possible during a session, and this element too requires careful consideration, and might need to be addressed during the session itself.

It is essential that patient and therapist be alone, in a room, which should warrant privacy for the duration of the interview, without the possibility of a bystander accidentally overhearing or—worse—actually walking into the session. It might take longer to initiate the session, as both parties will have to adjust to the new setting, after a few minutes though, the discrepancy with the original session will weaken and possibly disappear entirely if the parties have already established a solid working alliance.

A further important issue concerns payment for the sessions. We have frequently heard of therapists that, due to themselves or their patient relocating, continue therapy online requesting a lower fee. From a systemic perspective, every action constitutes an important communication, so charging less for an online session than for a face-to-face one automatically discounts the value of the session itself. The patient will receive the message that 45 or 60 online minutes are worth less than 45 or 60 min live. From that moment onwards, treatment will be discounted as a second rate work. It is possible that therapists feel as a loss, believing that they cannot provide the same quality of relationship online that they would in their usual setting; this is especially true if therapists feel that an online session will require less commitment, attention or participation than a live session. For all the above reasons, it is essential for the therapist to carefully consider whether it is appropriate to continue an online treatment or, given the financial and relational discounting of the process, if it wouldn’t be best to refer the patient to another therapist.

Just as is customary for normal sessions, appointments for the next online session must be decided at the end of the previous one; some aspects that must be taken into account for long-distance sessions are time differences and the necessary conditions of privacy for both patient and therapist.

Given that today’s technology allows one to host a video chat not only from a desktop computer but also through the use of smartphones or tablets, it is important that the therapist does not improvise the location of the session. Form and formalities must be respected in order to replace the privacy, continuity and reserve usually provided by the therapist’s office.

## An Example of Therapy via Video Conference

Consent given by the client for an edited version of name and data of his story.

Nicolò is a 33-year-old handsome young man, fashionably dressed in casual designer clothes; he is referred to treatment by his mother. He complains of depressive aspects, feeling unstable, and most importantly a difficulty in making decisions, which his profession requires of him. He was born in a low-income district and spent much time on street corners from the age of 6, when his parents divorced. He has undertaken many different kinds of activity (waiter, sales assistant, deliveryman, party organizer…) until he stumbled onto his real vocation: he became an event organizer and a very efficient manager, founding his own company and promoting his products through fantastic and well made videos. He is proud to underscore that, unlike many of his childhood friends from the same low-income area, who ended up being involved with drugs and crime, he was able to transform himself into a really successful manager, troubled only by some persistent and incomprehensible moments of insecurity and anxiety. While his mother lives in Italy, in a home that he bought for her, he spends most of his time on a Greek Island, where he has recently opened another branch of his company, originally and still based in Croatia. He lives with his girlfriend, of whom he really appreciates both professional and personal qualities; she is also his primary assistant and collaborator. The life they lead, based on seasonal work that keeps him busy mostly at night, between parties and events that go on until dawn, does create some tension in their otherwise affectionate and collaborative relationship, which is having a hard time becoming more solid.

Exploring the events surrounding the opening of his new branch, which coincided with the emergence of his symptoms, it surfaced that in Croatia he was strongly pressured by a powerful business competitor who wanted to take over his business, and this led Nicolò to relocate his resources to another region, where there was less competition. This however was not sufficient to reduce the feeling of instability he felt pervaded by, and he developed the fear that the competitor would pursue him to the new location.

After the initial sessions, in which we explored the fear of going back from being a budding star in the tourist economy to falling back into the boy from the low-income neighborhood, we began working on reconstructing his self-esteem and providing psychological support. Nicolo has to return to the Greek island, and from then onwards it becomes progressively more difficult to meet him in the therapist’s cabinet, it becomes necessary to schedule 40-minute sessions via a videoconference tool, initially every 15 days, then once a month.

During the first of these online meetings, the therapist is at home, behind him hangs a poster of a conference—a copy of which also hangs in his study at work. He is wearing his formal work attire, suit and tie. Nicolo is wearing an old dirty t-shirt; he is unshaven, sitting at his 1970s kitchen table, a pasta strainer hanging behind him. The therapist makes a comment about the difference with the young man’s usual appearance and learns that the furniture is the same that Nicolo grew up with in his mother’s home, which he took to Greece when he moved. This leads Nicolo to open up about his fears; he is afraid of the failure, which would send him back, unemployed, to that poverty-stricken neighborhood; he is afraid of success, which would separate him even more from that world that he feels he belongs to, and—possibly—from his mother with whom he grew up. He constantly lives this division between the fear of going back and the fear of going too far forward, and he distances himself from these fears by ascribing them to the aggressive persecution and competition on behalf of his rival. At the same time, this persistent insecurity, with its’ periodic symptomatic manifestations, justifies him in not fully separating from his mother and from moving forward and creating his own family. In fact, his happy cohabitation with his girlfriend is periodically interrupted by break-ups during which he returns to his mother’s house.

Therapy continues with excellent results, with a ratio of two or three online conferences per every face-to-face session. The use of video conferences allowed Nicolo to show the therapist his hidden face, and the therapist was able to intervene on a personality issue early in the process: the young man’s difficulty in becoming a fully-grown adult, hindered by his old fears of financial and emotional loss.

## Therapists and Social Networks

We know, to this day, that the therapist’s profession is a not very “social” profession: for instance, it is not recommendable for a therapist to link with patients via Facebook or Instagram, and it’s better that his social network’s profile maintain an high credibility and an high privacy level (could you imagine the reaction of your patients finding online a photo of you in bikini, or having fun during a party in high school?). It is becoming increasingly difficult to remain outside of the social networking world given that the latter has, somehow, wormed itself into the lives of everyone. By “social network” we refer to all the software and platforms which favor the construction of virtual networks and connections enabling users to connect with each other and share all sorts of digital content (text, audio, video, etc). Usually social networks require some form of registration and the creation of a password protected personal profile; it is also usually possible to search these networks to locate and identify other users and to organize them in groups. Shared information may vary from network to network; they may include personal data (religious orientation, political affiliation, sexual preferences, etc.) and professional data.

Social networks not only use content, but they can generate content; in this sense, the web becomes an interactive environment through which one can divulge ideas, links, and multimedia content.

Because of the widespread use of social networks, sociologists have defined this the era of the person-to-person network (Rainie and Wellman 2012): social exchanges no longer occur exclusively inside predetermined systems such as family, groups, neighborhoods, but are intensely lived with individuals who become part of a person’s network.

How can we manage our online identity without repercussions on our offline relationships and on the therapy setting?

Concerning professional networking systems, such as LinkedIn, the user is assisted in filling in his or her profile, by adding only the professional information; these social networks become important tools to gather, in a single place, all the information we wish to make public about ourselves. Like blogs or personal websites, these networks allow users to control the information posted online and to manage their online identity with the aim of controlling information that may become inconvenient if public. One of the authors of this paper, for example, has a homonymous peer who is a professional singer and has a very active YouTube page. This discovery was made during the first sessions with a patient who, being a big fan of karaoke, peppered the entire interview with comments and sidejokes about singing (comments along the lines of “*we have a shared understanding Dr*.”) which the therapist could not fully understand. After having made this explicit during the second session, the patient revealed that he had googled the therapist and—having found the singer’s youtube page—had assumed that this was the same person leading a double life (suit and ponytail by day; glitter and pink wig by night). So much did the patient want to be accepted and welcomed that before even starting the therapy he had already identified these deep connections with the therapist!

We believe that for this very reason it is important for therapists to have a virtual space (a Facebook page, a blog, a LinkedIn profile) of which they can control content and diffusion.

Concerning social networks like Facebook, a public page could be the means for a therapist to gain more visibility, if the content published is meaningful. Most therapists’ pages provide limited information and standard phrases, which have little to do with their profession: “You can only see things clearly with your heart. What is essential is invisible to the eye.” is one of the typical quotes that can be read on a therapist’s public profile, and in fact, it is one of the most quoted lines from The Little Prince (de Saint-Exupéry 1943).

Concerning the management of a private profile, this should not present a problem if the therapist keeps a high level of privacy; still, it is good practice to be careful when posting profile pictures.

Another significant problem is the network of online friends. It is highly possible that, once being given the contact information, potential patients will look up the therapist’s information online, and it is possible that in viewing a therapist’s Facebook profile they may discover a common friend. This is not exclusive of online relationships as it can happen in real life as well, but in some cases, it could lead to situations, which need to be discussed in therapy. One example is that of a patient who, having viewed her therapist’s Facebook page discovered that they had a common friend with whom the patient had severe issues both of a professional and financial nature. This led the patient to open up during the second session with the therapist and say “*If I choose to come here I need to know that I can talk of whatever I need to discuss. The fact that you and X are friends on Facebook makes me wonder if I can talk to you about the very big problems I am having with X*.” This opening actually turned out to be a very important moment towards the establishment of the therapeutic alliance and of a therapy contract.

## Conclusions

Psychotherapy is the last of the magic arts, through which humans try to change themselves and others, and the reality of their world by using themselves and their words. The cybernetic world seems to testify to the triumph of science, a world in which every individual can find, through an App, the solution to his or her psychological or life problem.

This is in fact a non-existent juxtaposition: the fact that sessions take place in a face-to-face setting does not warrant their effectiveness, nor does the existence of a thousand self-help Apps testify to their utility (if they did indeed work, there would be fewer).

Magic avails itself of technical tools, such as magic wands, amulets, smoke and mirrors, and any progress made in the development of these tools can simplify work for magicians and their clients. Technology can help all of us to gain some trust, even as just a placebo effect, when there are small obstacles and we can gain some reassurance from these tools. We do believe that new communication technologies can be used to integrate face-to-face treatment, but they cannot replace it.

Sherry Turkle, professor of Social Studies and Technology at MIT, shares our position; Turkle was concerned by the observed 40% drop over the last 30 years of the student’s empathic capacities and this was ascribed, in a research conducted at the University of Michigan (Konrath et al. 2011), to the development of new technologies. In truth, Turkle states, psychotherapists “are expert in that kind of conversation which the digital culture needs the most: the kind of exchange that is more interpersonal than transactive” (Turkle 2016).

It is impossible to foresee the incredibly rapid developments of technology; in the 1920s a technician from an automatic piano company, which boasted an instrument that could reproduce 16 degrees of dynamic intensity of sound, was rejected by the great pianist Artur Schnabel who stated “Unfortunately, I use seventeen” (Gaisberg 1947).

The variety of interactions, which a human can generate, despite errors or imperfections, will always be superior to programs and protocols, which limit expressivity in live concertos as they do for therapy. That interpersonal quality which is a special extra added by face-to-face meetings is always going to be a step ahead of the possibilities provided by a programmed reproduction.
